# Hierarchically Ordered Supramolecular Protein-Polymer Composites with Thermoresponsive Properties

**DOI:** 10.3390/ijms160510201

**Published:** 2015-05-05

**Authors:** Salla Välimäki, Joona Mikkilä, Ville Liljeström, Henna Rosilo, Ari Ora, Mauri A. Kostiainen

**Affiliations:** 1 Biohybrid Materials Group, Department of Biotechnology and Chemical Technology, School of Chemical Technology, Aalto University, 00076 Aalto, Finland; E-Mails: salla.valimaki@aalto.fi (S.V.); joona.mikkila@aalto.fi (J.M.); ville.liljestrom@aalto.fi (V.L.); ari.ora@aalto.fi (A.O.); 2 Molecular Materials Group, Department of Applied Physics, School of Science, Aalto University, 00076 Aalto, Finland; E-Mail: henna.rosilo@aalto.fi

**Keywords:** block copolymer, ferritin, dendron, protein cage, stimuli-responsive, self-assembly, biohybrid material

## Abstract

Synthetic macromolecules that can bind and co-assemble with proteins are important for the future development of biohybrid materials. Active systems are further required to create materials that can respond and change their behavior in response to external stimuli. Here we report that stimuli-responsive linear-branched diblock copolymers consisting of a cationic multivalent dendron with a linear thermoresponsive polymer tail at the focal point, can bind and complex *Pyrococcus furiosus* ferritin protein cages into crystalline arrays. The multivalent dendron structure utilizes cationic spermine units to bind electrostatically on the surface of the negatively charged ferritin cage and the *in situ* polymerized poly(di(ethylene glycol) methyl ether methacrylate) linear block enables control with temperature. Cloud point of the final product was determined with dynamic light scattering (DLS), and it was shown to be approximately 31 °C at a concentration of 150 mg/L. Complexation of the polymer binder and apoferritin was studied with DLS, small-angle X-ray scattering, and transmission electron microscopy, which showed the presence of crystalline arrays of ferritin cages with a face-centered cubic (fcc $Fm\bar{3}m$) Bravais lattice where lattice parameter *a* = 18.6 nm. The complexation process was not temperature dependent but the final complexes had thermoresponsive characteristics with negative thermal expansion.

## 1. Introduction

Polymer-directed protein assemblies have emerged as important class of biohybrid materials [1]. For example biomedical systems that combine highly versatile synthetic polymers with the precisely controlled assembly properties of native proteins can achieve intriguing new properties, such as prolonged duration of action [2], protection against degradation [3], enhanced delivery [4,5] or stimuli-responsiveness [6,7]. Covalent modification of proteins with synthetic polymers has been studied intensively during the past years [8,9]. Three ways to create such conjugates are commonly utilized: grafting-from, grafting-to and grafting-through. Examples of such conjugates include for example: oligo(ethyleneglycol methacrylate) on salmon calcitonin or trypsin [10,11], alkyne modified poly(ethylene glycol) clicked on azide derivatized protein [12], and Pluronic-fibrinogen based hydrogels [3]. Especially temperature sensitive protein-polymer conjugates have received focused attention due to their ability to respond to external stimuli and modify for instance enzyme activity [13]. However, supramolecular binding of polymers on native proteins has been less studied [14,15,16]. Furthermore, supramolecular composite structures that exhibit nanoscale order and consist of polymers and proteins are only starting to emerge [17,18,19,20,21].

Dendrimers are branched treelike molecules with unique properties [22,23]. They have a well-defined structure, a monodisperse size distribution, and their solution properties are mainly defined by the surface groups. Multiple surface groups allow the construction of multivalent arrays of binding ligands that can achieve high-affinity towards biomolecules [24]. We have previously presented cationic spermine-functionalized dendrimers that can bind biomolecules, such as DNA and, importantly, also native proteins with high affinity [25,26,27]. We have been especially interested in developing dendrimers that bind and pack protein-based nanoparticles (viruses and ferritins) into crystalline structures [28,29,30,31,32].

Ferritin is a round hollow protein assembly found in most living organisms and its major function is to host iron in ferrihydrite form. Ferritin cage has an outer diameter of 12 nm and inner diameter of 6 to 8 nm. The empty ferritin cage is called apoferritin (aFT) and is formed of 24 protein subunits. Like other nanocages [33,34,35], ferritin can also be exploited as a nanocarrier [36,37]. Its inner cavity can be used as a storage for therapeutics or imaging agents and its outer layer may be modified to enhance targeting [38,39]. Ferritin has also been used in contrast enhancement for magnetic resonance imaging [40,41]. Because ferritin has a low relaxivity, it must be enhanced before it reaches the levels needed for efficient contrast enhancement. This can be done with controlled aggregation [32,42] or with addition of enhanced MRI agents [43]. Moreover, magnetoferritin with a superparamagnetic iron oxide core has been utilized [44]. Since interior of ferritin is sequestered from the outer environment, ferritin’s inner cavity could be used also as a reaction vessel for catalytic reactions. For example, platinum (Pt(0)) has been synthesized inside aFT and further used to catalyze hydrogenation of olefins [45]. In addition, protein cages could be used in electronic devices. Especially, the ability to control magnetic properties makes ferritin attractive for further development of electronic applications [46,47,48].

In this work, the electrostatic binding of anionic aFT and cationic thermoresponsive spermine dendrimer is studied in order to develop water soluble and temperature-controlled self-assemblies held together by supramolecular interactions. Ferritin from *Pyrococcus furiosus* has isoelectric point between 4.5 and 5.5, indicating a net negative charge in neutral and basic environment. Correspondingly, below isoelectric point ferritin has a net positive charge. Charged ferritins attract oppositely charged particles, which leads to electrostatic complexation if the attraction is high enough. The assembly and final nanostructure of higher-order structures is dependent on strength of the electrostatic interactions. Synthesis of spermine dendron is achieved using standard peptide coupling reactions and orthogonal protecting group strategy. Polymerization of poly(di(ethylene glycol) methyl ether methacrylate) linear block is carried out using atom transfer radical polymerization (ATRP). Dynamic light scattering (DLS), small-angle X-ray scattering (SAXS) and transmission electron microscopy (TEM) are used to show that the two components interact electrostatically and self-assemble into crystalline complexes, where the periodicity can be tuned with temperature.

## 2. Results and Discussion

### 2.1. Synthesis

**Figure 1:**
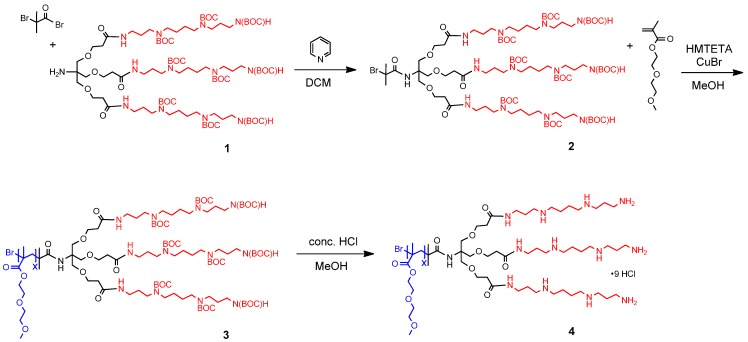
Synthesis of the stimuli-responsive linear-branched diblock copolymer (**4**). The branched part consists of a cationic multivalent dendron with a trifurcated Newkome-type frame and spermine (**red**) functionalized surface. The linear thermoresponsive polymer tail (poly(di(ethylene glycol) methyl ether methacrylate)) (**blue**) at the focal point is polymerized *in situ* using atom-transfer radical polymerization.

The synthesis of the target compound **4** (Figure 1) was initiated by preparing a first generation trifurcated Newkome-type dendron with a *tert*-butyloxycarbonyl (BOC) protected spermine surface and a free amine group at the apex (compound **1**, synthesis has been reported previously [31]). The dendron was synthesized utilizing divergent step synthesis and purified by silica column chromatography. A bromoisobutyryl bromide polymerization initiator was attached to the core via a peptide bond and base catalysis to yield macroinitiator **2**.

Di(ethylene glycol) methyl ether methacrylate (DEGMA) was polymerized with atom transfer radical polymerization *in situ* to the dendron and purified by dialysis. The polymerization was carried out by first purging separately a MeOH solution of 1,1,4,7,10,10-hexamethyltriethylenetetramine (HMTETA) and CuBr with nitrogen gas for 20 min and then combining them under nitrogen atmosphere. HMTETA was added to the CuBr with a two-ended needle and 1 mL of the gained solution was added to a nitrogen purged flask. DEGMA and the macroinitiator **2** were as well purged with nitrogen for 15 min and then added to the HMTETA/CuBr solution. Reaction mixture was stirred for 50 h at 50 °C. Product **3** was isolated by successive dialysis against methanol and water. Removal of the BOC groups was achieved by acid hydrolysis in a 1:4 mixture of concentrated HCl and MeOH. Solvents and BOC hydrolysis products were removed using reduced pressure to obtain the target compound as a light yellow wax.

Reactions were monitored with thin layer chromatography (TLC) and proton nuclear magnetic resonance (^1^H NMR) spectroscopy, which confirmed the structure of the target compounds. For the final product, **4**, the average length of the polymer tail was defined based on the ^1^H NMR spectrum and was found to be eight repeating units, giving a final molecular weight of 2502.98 g/mol. Overall, the polymer tail is short when compared to the initial amount of monomer due to the unoptimized polymerization conditions and likely steric effects caused by the bulky dendritic part (see the Supplementary Information Materials and Synthesis sections for full details).

### 2.2. Self-Assembly of Large Protein-Polymer Complexes

**Figure 2:**
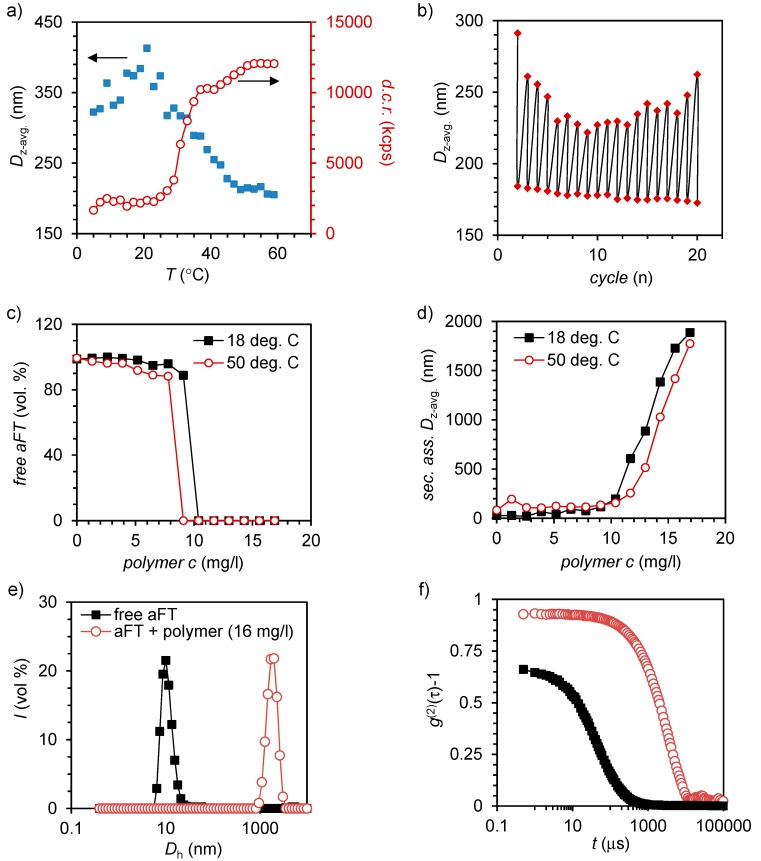
(**a**) Z-average diameter and derived count rate for **4** (150 mg/L) as a function of temperature; (**b**) Reversible thermal switching of size by cycling temperature between 18 and 50 °C; (**c**,**d**) Titration of aFT with **4** below (18 °C) and above (50 °C) *T*_cp_ followed by the scattering from free aFT (**c**) and formation of large secondary assemblies (**d**,**e**). Volume-average size-distribution measured from free aFT (100 mg/L) and aFT-**4** (16 mg/L) complexes; and (**f**) the corresponding second-order autocorrelation curves.

DLS was used to determine properties of polymer **4** and its electrostatic complexes with aFT (Figure 2). Variable temperature DLS measurements were first used to measure the cloud point (*T*_cp_) for polymer **4** alone. Cloud point measurements were carried out by measuring the hydrodynamic diameter (*D*_h_) and derived count rate (d.c.r.) on a chosen temperature range (5–59 °C) (Figure 2a). Clear changes in the derived count rate were observed as a function of temperature. The count rate was observed to increase with increasing temperature and decreasing size. At 5 °C the d.c.r. is approximately 200 kilo counts per second (kcps) and it stabilizes at 9000 kcps when approaching 60 °C. The *T*_cp_ based on the count rate profile is centered at 31 °C, which matches well with the values reported for similar DEGMA chains coupled to cationic polymers [18].

For a sample with a concentration of 150 mg/L, z-average size decreases from ~350 to ~200 nm upon heating, indicating polymer chain collapse. Most likely, the collapse of the relatively short DEGMA chain does not result in the formation of large clusters and the polymer chains remain relatively free in the solution and therefore the average size is observed to decrease. The transition occurs approximately between 25 and 45 °C, which matches with the observed transition in the d.c.r. This change in temperature is reversible and can be cycled multiple times by changing between two temperatures, 18 and 50 °C (Figure 2b). However, it must be noted that due to the relatively low molecular weight and low scattering intensity of compound **4**, the measured hydrodynamic sizes should be taken as indicative only. However, the temperature-responsive characteristic of the compound is clearly observed.

The ability of polymer **4** to bind and complex electrostatically native proteins in aqueous solutions was studied by titrating an aFT solution with polymer **4**. Titration was done in two temperatures, 18 and 50 °C, to see how temperature affects complexation. Previous studies have indicated that the temperature and polymer chain conformation can play a major role in how efficiently the complexes are formed [18]. Formation of complexes and amount of free aFT were observed with DLS. First, aFT solution of 750 μL volume and 100 mg/L concentration was prepared and then titrated with the polymer **4** solution (0.5–2.5 mg/mL). Titration indicated that temperature does not have a significant impact on complexation (Figure 2c). At 50 °C, a polymer concentration of 9.1 mg/L was enough to complex all aFT. At 18 °C, 10.4 mg/L of **4** was needed for full complexation. Difference is negligible as at both temperatures roughly one tenth of the aFT concentration was enough to bind all free aFT. The scattering peak corresponding to aFT disappears when more polymer is added and larger, secondary, complexes start to form. In both temperatures, at the last titration point the size of the complexes is approximately 2000 nm in diameter (Figure 2d). A comparison of volume-average sizes corresponding to free and complexed aFT solutions is presented in Figure 2e, which shows that uniform secondary complexes are formed whereas free aFT disappears. Figure 2f shows the corresponding second-order autocorrelation curves. In addition, ζ-potential of aFT, **4** and different mixtures of them were measured. As expected, the measured ζ-potential value for aFT was negative (−21.70 mV) and for **4** positive (7.59 mV). By increasing the polymer concentration to 50 mg/L or slight excess (*w*/*w*), the ζ-potential of aFT can be increased gradually to 4.4 mV (see the Supplementary Information Figure S1). 

Similar complexation behavior of ferritin has been observed also previously. For example, magnetoferritin has been complexed with light-responsive low generation dendrimers with spermine tails [29]. In these studies analogous secondary complexes were formed as the scattering originating from free aFT particles was reduced. Additionally, these complexes could be disassembled with low-intensity UV irradiation. Also, other negatively charged protein cages have been used to form complexes with oppositely charged substances, such as virus particles. Cowpea chlorotic mottle virus (CCMV) is one of the extensively studied protein cages with negative surface charge. It has been complexed, for example, with Janus-dendrimers [31] and thermoresponsive polymers [18] with formation of same kind of complexes as observed in this study.

### 2.3. Nanostructure of the Crystalline Protein-Polymer Complexes

**Figure 3:**
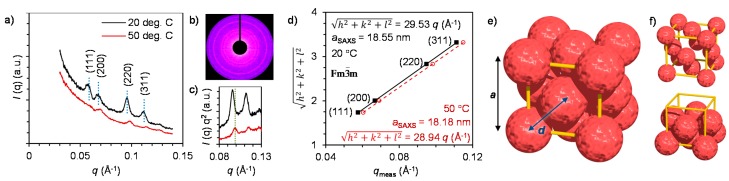
(**a**) Azimuthally integrated SAXS profiles for aFT-**4** complexes measured at 20 and 50 °C. Dotted vertical lines indicate the calculated peak positions for a fcc structure with *a* = 18.55 nm; (**b**) 2D scattering profile of the 20 °C sample; (**c**) Comparison between the (220) and (311) peak positions measured at different temperatures highlights the change in lattice constant; (**d**) Quadratic Miller indices of assigned reflections for fcc structure *versus* measured *q*-vector positions for indexed peaks. Lines present linear fits, which yield lattice parameters 18.55 and 18.18 nm at 20 and 50 °C, respectively; (**e**) Face-centered cubic (fcc) unit cell (yellow) of aFT particles (red) drawn to scale; and (**f**) unit cell with aFT diameter reduced for clarity (top) and unit cell with top 5 aFT removed.

Crystalline ordering of individual aFT particles was studied by SAXS and TEM. We have recently established that approximately spherical protein cages can be assembled into crystalline assemblies by carefully controlling the strength of electrostatic interactions in the aqueous system by the addition of electrolytes [19,29]. Similar behavior was also observed with the current aFT-**4** system. In the absence of added electrolyte (NaCl) the complexes exhibit an amorphous structure that is a result from a rapid kinetically trapped assembly pathway. When the NaCl concentration is increased to 10 mM, the electrostatic interactions are partly screened, kinetic traps can be avoided and crystalline assemblies are formed. At even higher NaCl concentrations, the electrostatic attraction between the polymer and aFT are reduced to such an extent that only free aFT particles are observed. The crystallographic arrangement of aFT particles in the crystalline samples (10 mM NaCl) was first studied by SAXS at 20 °C. Figure 3a shows azimuthally integrated SAXS profiles where a distinct scattering pattern can be observed. The original 2D scattering profile for the 20 °C sample is displayed in Figure 3b. Bragg reflections from the (*hkl*) = (111), (200), (220) and (311) planes are observed at *q* = 0.0605, 0.0692, 0.0983 and 0.11491 Å^−1^, respectively. For cubic lattices the lattice constant can be calculated as: *a*_saxs_ = 2π√(h^2^ + k^2^ + l^2^)/*q*(hkl), and determined by plotting the measured *q*(hkl) values against the quadratic Miller indices with a linear regression fit to give q* = 0.03386 Å^−1^ and *a* = 18.55 nm. Based on the peak positions and quality of the fit, the scattering pattern can be assigned to a face-centered cubic (fcc) Bravais lattice with space group $Fm\bar{3}m$ , number 225 (Figure 3e,f). Here, the center-to-center distance of aFT particles is *d*_aFT-aFT_ = *a*/√2 = 13.1 nm, which corresponds well with the dimensions of the aFT cage covered with a polymer layer.

Changes in the crystal structure as a function of temperature were studied by heating the sample above the *T*_cp_ of the polymer to 50 °C. Collapse of the polymer chain at elevated temperatures is expected to result in smaller lattice constant as the volume occupied by the polymer chain is reduced and consequently the aFT particles can pack closer to each other. Indeed, increasing the temperature was observed to distort the crystal to yield less pronounced scattering peaks, but also to shift the peaks to higher *q*-values indicating the formation of a more compact crystal structure (Figure 3c). The structure at 50 °C can also be indexed to an fcc structure, but with a smaller lattice constant *a* = 18.18 nm (Figure 3d). Although the chance in the lattice constant is small, it could be possible to increase the effect by using a polymer with a higher molecular weight. Importantly, the current results establish that the dimensions of the unit cell can be affected by temperature, giving the aFT-**4** crystal a negative thermal expansion in the studied temperature range. Similar approximately few nanometer scale changes have been achieved with for example DNA coated nanoparticles [49].

**Figure 4:**
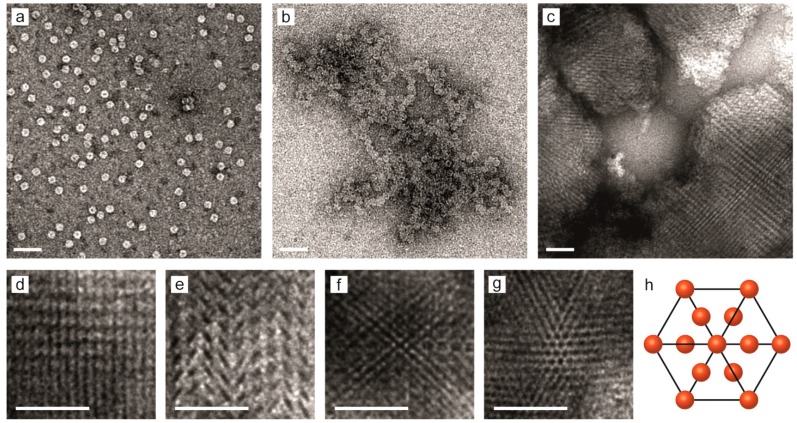
Negatively stained (uranyl acetate) TEM images of: (**a**) Free aFT particles (**b**) aFT-**4** complexes in the absence of NaCl are large and amorphous; (**c**) aFT-**4** complexes prepared in the presence of 10 mM NaCl show large crystalline domains; (**d**–**g**) Crystalline assemblies viewed along different projection axes; and (**h**) fcc unit cell viewed along the [111] zone axis. Scale bars are 50 nm in all images.

Samples with the three expected morphologies: amorphous colloidal glass, crystalline and free particles were imaged with TEM. aFT particles without any added polymer **4** were imaged after negative staining with uranyl acetate. The particles show the expected spherical shape with an average diameter of approximately 12 nm (Figure 4a). When polymer **4** is added in the absence of NaCl, large amorphous aggregates with diameters of several hundreds of nanometers can be clearly observed (Figure 4b). The results correspond well with the DLS measurements. In the samples with 10 mM NaCl concentration, ordered crystalline arrangement can be observed in agreement with the SAXS results. Figure 4c shows a low-magnification of image of several crystalline assemblies viewed along different projection axes. A collection of the crystalline assemblies viewed along different projection axes is presented in Figure 4d–g. Figure 4g shows a crystal projection viewed along the [111] zone axis, which is hexagonal for a fcc Bravais lattice (the (111) plane is 2D hexagonally-close packed). A comparison to a schematic unit cell viewed along the [111] zone axis is presented in Figure 4h.

## 3. Experimental Section

### 3.1. Dynamic Light Scattering 

DLS instrument (Zetasizer Nano Series, Malvern Instruments, Worcestershire, UK) equipped with a 4 mW He-Ne ion laser at a wavelength of 633 nm and an Avalanche photodiode detector at an angle of 173° was used to measure both the hydrodynamic radius diameter and the electrophoretic mobility. The measurements were carried out in Plastibrand PMMA cuvettes (BrandTech Scientific, Essex, NJ, USA). Zetasizer software (Malvern Instruments) was used to obtain the scattering intensity (count rate), particle size distributions and the electrophoretic mobility. 

### 3.2. Small-Angle X-ray Scattering 

The samples were prepared by adding 1.25 µL NaCl water solution in 5 µL aFT solution (10 mg/mL) to adjust the ionic strength after which 3.75 µL polymer **4** (10 mg/mL) was added. The liquid samples were sealed between two Kapton films during the SAXS measurements and the sample environment was evacuated to reduce scattering from air. The SAXS was measured using a rotating anode Bruker Microstar microfocus X-ray source (Cu Kα radiation, λ = 1.54 Å, Bruker, Madison, WI, USA). The beam was monochromated and focused by a Montel multilayer focusing monochromator (Incoatec, Geesthacht, Germany). The X-ray beam was further collimated by a set of four slits (JJ X-Ray, Lyngby, Denmark) resulting in the final spot size of less than 1 mm at the sample position. The scattered intensity was collected using a Hi-Star 2D area detector (Bruker). Sample-to-detector distance was 1.59 m and silver behenate standard sample was used for calibration of the length of the scattering vector *q*. One-dimensional SAXS data were obtained by azimuthally averaging the 2D scattering data. The magnitude of the scattering vector *q* is given by *q* = 4π(sin θ)/λ, where 2θ is the scattering angle.

### 3.3. Transmission Electron Microscopy Imaging 

Polymer-aFT samples were prepared by combining 2 µL of aFT water solution (1 mg/mL) with 3 µL of polymer water solution (0.1 mg/mL), both having the desired electrolyte concentration (0, 10 or 15 mM) and then diluting the gained sample with 13 µL of NaCl solution with a corresponding electrolyte concentration. Imaging was carried out with Tecnai 12 Bio-Twin transmission electron microscope (FEI, Hillsboro, OR, USA) using an acceleration voltage of 120 kV. Sample volumes of 3 µL were placed on the grids and left there for 2 min, after which the excess liquid was blotted away with filter paper. The samples were imaged both with and without uranyl acetate (negative) staining. Formvar carbon on 400 mesh copper grids (PSI supplies) or Carbon film on 300 Hex Mesh copper grids (Electron Microscopy Sciences, Hatfield, PA, USA) were used. Grids for samples with added NaCl were plasma cleaned before sample preparation.

## 4. Conclusions

We have synthesized Newkome-type spermine dendron with ATRP initiator in the focal point. *In situ* polymerization of di(ethylene glycol) methyl ether methacrylate from the dendrimer was also achieved. The final product was water-soluble and thermoresponsive, with *T*_cp_ of 31 °C. Furthermore, the transition at the cloud point was shown to be reversible. The polymer’s ability to bind and pack aFT protein cages into hierarchically structured complexes was also studied. Complexation was based on electrostatic attraction between the negatively charged aFT and cationic dendrimer. aFT was shown to be packed into micron-sized complexes with increasing polymer concentration. Complexation did not show clear dependence on temperature. However, complexes were to some extent thermoresponsive, showing a negative thermal expansion. ζ-potential of these complexes was shown to be dependent on the dendrimer concentration, and therefore tunable.

When the electrostatic interactions between the polymer and aFT are tuned with salt to a weakly attractive regime, crystalline protein-polymer composite assemblies can be formed. Face-centered cubic structure with lattice parameter *a* = 18.55 nm was observed with SAXS. TEM imaging confirmed the presence of large crystalline assemblies. The lattice constant was found to reduce to 18.18 nm when the crystalline complexes were heated above the *T*_cp_ of the polymer. Advantages of this system lay in the non-covalent assembly of particles and in the possibility to modify charge and size of the complexes. Thermoresponsive properties of the complexes should be optimized to achieve crystal structures where the lattice constant can be tuned to a larger extent.

## Supplementary Materials

Supplementary materials can be found at http://www.mdpi.com/1422-0067/16/05/10201/s1.
