# Supplementary material for: Hierarchically Ordered Supramolecular Protein-Polymer Composites with Thermoresponsive Properties
Source: Int J Mol Sci. 2015 May 5;16(5):10201–13. doi: 10.3390/ijms160510201 (PMC4463641; doi:10.3390/ijms160510201)
Supplement: Supplementary file 1 [file ijms-16-10201-s001.pdf]

Supplementary Information

1. Materials

Di(ethylene glycol) methyl ether methacrylate monomer was purchased from Aldrich and it was purified by eluting trough Al_2O_3 with methanol. Hydroxybenzotriazole (HOBt), *N*,*N*-dicyclohexylcarbodiimide (DCC), triethylamine (Et_3N), piperidine, bromoisobutyryl bromide (BIBB), pyridine, Na_2SO_4, NaCl, 1,1,4,7,10,10-hexamethyltriethylenetetramine (HMTETA), CuBr, tris(hydroxymethyl) aminomethane (Tris) and HCl (32%) were all purchased from Aldrich and used without further purification. Silica and ninhydrin were purchased from Fluka and used as received. Apoferritin (aFT) was purchased from Molecular Links Rome (Rome, Italy) and used without purification. Ninhydrin stain was prepared by dissolving 1.5 g ninhydrin in 100 mL of *n*-butanol and 3 mL of acetic acid. Dichloromethane (DCM), tetrahydrofuran (THF), dimethylformamide (DMF) and methanol (MeOH) were purchased from Aldrich.

2. Synthesis

*2.1. Synthesis of Compound **2***

Compound **1** (254 mg, 0.14 mmol) was dissolved in anhydrous DCM (15 mL). Bromoisobutyryl bromide (65 mg, 0.28 mL) and pyridine (27 mg, 0.34 mmol) were added. Reaction mixture was stirred in room temperature for 45 h. Small amount of both BIBB and pyridine were added an hour before stopping the reaction. Crude product was purified by extraction with brine twice (20 mL). Gained water phase was then extracted twice with DCM (20 mL) and organic phases were combined and dried with Na_2SO_4. The solids were filtered out and DCM was evaporated under reduced pressure. Final purification was done with gel column chromatography (Biobeads, DCM/MeOH 9:1) and with silica column chromatography (DCM/MeOH, 95:5). The product was obtained as a yellow wax (74.4 mg, yield 28%):

TLC R_f = 0.3 (DCM:MeOH 95:5, ninhydrid stain)
^{1}H NMR (400 MHz, $CDCl_3$): δ (ppm) 1.36–1.52 (81 H, $(CH_3)_3$C; 12 H, $NCH_2CH_2CH_2CH_2N$), 1.65 (12 H, $NCH_2CH_2CH_2N$), 1.83 (6 H, $C(CH_3)_2$), 2.46 (6 H, OCH_2CH_2CO), 3.04–3.30 (36 H, $CH_2CH_2CH_2N$), 3.61–3.80 (6 H, OCH_2CH_2CO; 6 H, CCH_2O).
IR: 3330m (NH); 2972, 2929s (CH_2); 1671s, (CON<); 1160s (R–CO_2–R)
MALDI-ToF MS$^+$: *m/z* calcd for $C_{92}H_{172}BrN_{13}O_{25}Na$ $[M + Na]^+$ 1963.3, found 1962.7.

*2.2. Synthesis of Compound **3***

First, a solution of HMTETA (3.7 mg/mL) and CuBr (1.5 mg/mL) in MEOH was prepared under nitrogen atmosphere. HMTETA in MeOH and CuBr were separately purged with nitrogen gas for 20 min. HMTETA was added to the CuBr with a two-ended needle. Nitrogen atmosphere was used to remove all air. One milliliter of this solution was added to a nitrogen-purged flask. DEGMA (400 mg, 2.13 mmol) and compound **2** (21 mg, 0.01 mmol) were as well purged with nitrogen for 15 min and then added to the flask. Reaction mixture was stirred for 50 h at 50 °C and conversion followed by NMR. NMR samples were taken with nitrogen-purged needles to avoid air contamination. Reaction was stopped by opening it to air and by removing it from oil bath. Copper was removed by filtrating the

solution trough silica (eluting with methanol). All colored copper compounds were absorbed into the silica. Part of the MeOH was removed with rotary evaporation and rest of the solution was purified with dialysis (MWCO 2000) against MeOH and water. Insoluble part of the mixture was separated by centrifugation and dried. Product was a light yellow solid (5.0 mg):

^{1}H NMR (400 MHz, DMSO): δ (ppm) 0.68–1.03 (45 H, OCOCCH_3), 1.30–1.42 (81 H, $(CH_3)_3$C; 12 H, $NCH_2CH_2CH_2CH_2N$), 1.56 (12 H, $NCH_2CH_2CH_2N$), 1.68–1.91 (7 H, polymer backbone CH_2), 2.27 (6 H, OCH_2CH_2CO), 3.04–3.16 (36 H, $CH_2CH_2CH_2N$), 3.19–3.30 (54 H, O(CH_3)), 3.40–3.49 (36 H, $CH_2OCH_2CH_2OCH_3$), 3.49–3.71 (64 H, $CH_2OCH_2CH_2OCH_3$; 6 H, OCH_2CH_2CO; 6 H, CCH_2O), 3.92–4.15 (30 H, $COOCH_2$).

*2.3. Synthesis of Compound **4***

Removing the BOC protecting groups of spermine was carried out by dissolving **3** (5.0 mg, 0.001 mmol) in 5 mL of MeOH. One and two-tenths milliliters of 32% HCl was added and the reaction was allowed to proceed overnight. Solvent was removed under reduced pressure and final product was lyophilized. Product was obtained as a light yellow wax (3 mg, 74% yield).

^{1}H NMR (400 MHz, D_2O): δ (ppm) 0.80–1.18 (21 H, OCOCCH_3), 1.75 (12 H, NCH_2 $CH_2CH_2CH_2N$), 1.88 (12 H, $NCH_2CH_2CH_2N$), 2.06 (8 H, polymer backbone CH_2), 2.52 (6 H, OCH_2CH_2CO), 2.96–3.18 (36 H, $CH_2CH_2CH_2N$), 3.19–3.31 (7 H, OCH_3), 3.31–3.48 (38 H, $CH_2OCH_2CH_2OCH_3$), 3.49–3.90 (80 H, $CH_2OCH_2CH_2OCH_3$; 6 H, OCH_2CH_2CO; 6 H, CCH_2O), 4.07–4.30 (16 H, $COOCH_2$).

3. ζ-potential

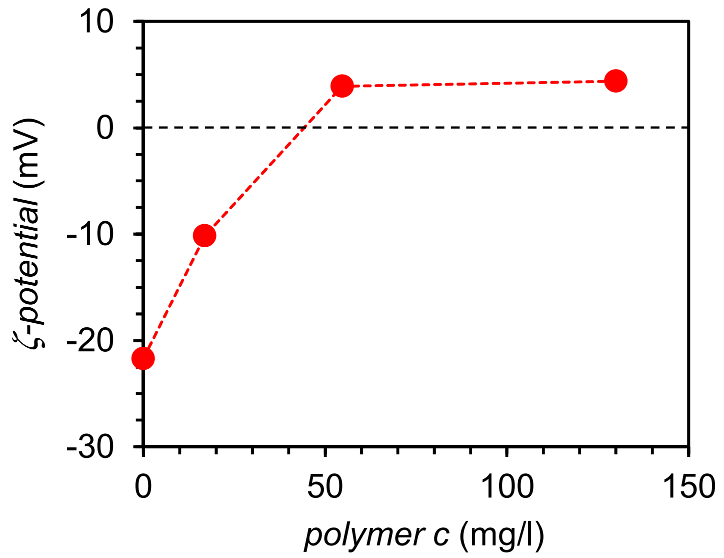

Figure S1. ζ-potential of aFT (100 mg/L) complexed with different amounts of polymer **4**. By increasing the polymer concentration to 50 mg/L or slight excess (*w*/*w*), the ζ-potential of aFT (−21.70 mV) can be increased gradually to 4.4 mV.
